# Pressure and Timing Differences Between Volitional and Non-Cued Saliva Swallows Measured with Pharyngeal High-Resolution Manometry

**DOI:** 10.1007/s00455-026-10988-7

**Published:** 2026-07-28

**Authors:** Maryann N. Krasko, Corinne A. Jones, Michelle R. Ciucci, Timothy McCulloch

**Affiliations:** 1 Department of Otolaryngology-Head & Neck Surgery, University of Wisconsin-Madison, 1300 University Ave, Madison, WI 53706, USA; 2 Department of Communication Sciences and Disorders, University of Wisconsin - Madison, 1975 Willow Drive, Madison, WI 53706, USA; 3 Department of Neurology, The University of Texas at Austin, 1601 Trinity St. BLDG B, Austin, TX 78712, USA; 4 Department of Speech, Language, and Hearing Sciences, The University of Texas at Austin, 1601 Trinity St. Bldg. B, Austin, TX 78712, USA; 5 Neuroscience Training Program, University of Wisconsin-Madison, 1111 Highland Ave, Madison, WI 53705, USA

**Keywords:** Swallowing, Deglutition, High-resolution manometry, Speech-language pathology, Pressure, Volitional swallowing, Spontaneous swallowing, Saliva swallow

## Abstract

Volitional and spontaneous swallowing occur regularly throughout the day, yet most research has primarily focused on cued volitional swallowing. Differences in sensory input and degree of cortical involvement between swallow types may contribute to differences in swallowing physiology. Pharyngeal high-resolution manometry provides an opportunity to characterize both swallow types. Pressure generation and timing events during cued volitional (2 cc thin liquid) and non-cued saliva swallowing were evaluated in 50 healthy adults (n = 25 per sex). Paired t-tests revealed that on average, significantly greater pressures were generated during volitional swallows than non-cued saliva swallows in the velopharynx (*p* < 0.0001), tongue base (*p* = 0.045), and UES (post-closure) regions (*p* = 0.0058), while no significant differences were noted in the hypopharynx (*p* > 0.05). Positive linear relationships between swallow types were identified for velopharyngeal and tongue base pressures. Additionally, volitional swallows demonstrated longer UES relaxation durations than non-cued saliva swallows (*p* = 0.0003). While C2-C4 size differed between males (larger) and females (*p* < 0.0001), sex was not associated with any swallowing outcome measures. Overall, healthy adults generated higher pressures during volitional than non-cued saliva swallows in most pharyngeal regions, whereas the hypopharynx appeared less influenced by swallow type. Positive relationships between swallow types suggest that volitional assessments can serve as useful proxies for spontaneous function and may provide clinically relevant information when standard bolus swallows are difficult to obtain. The absence of sex effects supports uniform interpretation of manometric results across adults. These results underscore the importance of considering swallow type when evaluating swallowing physiology and designing clinical interventions.

## Introduction

Oropharyngeal swallowing is a complex process, requiring precise neuromuscular coordination for the safe and efficient transfer of materials from the mouth to the esophagus. In terms of sensorimotor control, swallows can be considered volitional or spontaneous. Volitional swallows, which have been more widely studied, occur intentionally with the purpose of moving food, liquid, or saliva from the mouth to the esophagus for feeding [1]. Alternatively, spontaneous or non-cued saliva clearance swallows typically occur with minimal conscious awareness and serve to clear saliva and residual material to prevent airway compromise [1]. Although swallow frequency can vary within and between individuals due to various factors (e.g. meal consistency or meal size for voluntary swallowing; saliva production for spontaneous swallows), spontaneous swallowing likely occurs more frequently throughout the day (~ 1x/min in the awake state [2, 3] and ~ 3x/hour during sleep [4] compared to voluntary swallowing, which occurs primarily during meals. Prior neuroimaging studies suggest differences in neural activation patterns between volitional and spontaneous swallowing types [5–7]. For example, volitional swallowing has been associated with greater activation of the caudal anterior cingulate cortex and right insula than spontaneous swallowing [7]. These findings suggest that physiologic differences may also exist between these swallow types. Importantly, dysfunction in spontaneous swallowing could reduce the ability to clear secretions effectively, increasing the risk of aspiration and respiratory complications, and thereby having more adverse effects on overall health outcomes.

Despite this evidence, there is little research exploring the differences between these swallow types. This is largely due to limitations associated with examining spontaneous swallows with common tools. For example, videofluoroscopy relies on barium sulfate contrast material to visualize the bolus, which is not possible to administer for spontaneous swallows. Fiberoptic endoscopic evaluation of swallowing (FEES) can capture spontaneous swallows; however, because these events primarily clear saliva, they are often difficult to visualize due to the lack of contrast or food coloring. In addition, visualization of the bolus during FEES is limited by the location of the scope (e.g. velopharynx is not visualized once scope is in place), as well as by the “white out” period which occurs at the time of swallow. As such, swallowing research primarily stems from volitional swallow samples.

Volitional swallowing has been widely studied in a number of contexts and has been shown to vary among individuals due to external factors, such as bolus characteristics (*i.e*. bolus consistency, size, mass), as well as biological ones, such as body size and sex [8–22]. Males, for example, on average have a larger skeletal size [23], vertebral depth, vertebral height [24], and pharyngeal cross-sectional area [25, 26] compared to females. In the context of volitional swallowing, a change in a physical size variable, such as pharyngeal length, may also translate to an altered volitional swallow physiology, [1]. Similarly, sex has been shown to influence structural displacement (i.e. hyoid displacement, larynx-to-hyoid approximation) [27] and lingual pressure measures [28–30]; however, it may not play a significant role for temporal measures of swallowing [31]. While factors like size and sex have been shown to play a role in certain aspects of the volitional swallowing mechanism, again, their impact on spontaneous swallowing has not yet been described.

To address many of these limitations, pharyngeal high-resolution manometry (pHRM) can be used. pHRM is a useful tool for directly assessing pharyngeal pressures and determining pressure changes during swallowing throughout the pharynx and esophagus [1, 17, 19, 32–34]. The catheter, lined with 36 sensors roughly 1 cm apart, captures pressure as a function of space and time along the aerodigestive tract, yielding quantitative and reliable data. pHRM allows for the study of both volitional swallows via ingestion of different boluses, as well as non-cued saliva swallows that occur while the catheter is inserted in the nasopharynx throughout the duration of the study. This makes the tool well-suited for assessing subtle differences in pressure generation and duration among regions of the pharynx and between swallow types. Moreover, with evidence that factors such as sex and size can affect volitional swallowing, pHRM can be used to explore their potential effects on non-cued saliva swallowing, as well.

The primary aim of this study was to quantify differences between volitional and non-cued saliva swallows in healthy adults. Given prior evidence suggesting greater cortical involvement during volitional swallowing, we hypothesized that volitional swallows would yield greater pressures and durations compared to non-cued saliva swallows. Given that changes in pharyngeal pressures may be dependent on sex and pharynx size, secondary analyses included consideration of sex and vertebral size (as proxy for pharynx size).

## Methods

### Participants

This study included data from 25 male (age range 21–83) and 25 female (age range 21–89) participants. This study was completed with approval of the Institutional Review Board of the University of Wisconsin-Madison. Participants had no known swallowing or neurological disorders, no allergy to food and anesthetic materials, were not pregnant, and were tolerant of topical anesthesia for use in case of expressed discomfort.

### Equipment

All data was collected using a solid-state high-resolution manometer (ManoScan360 High-Resolution Manometry System, Medtronic, Minneapolis, MN), with a manometric catheter of 2.75 mm in diameter and 36 circumferential pressure sensors, each 2.5 mm wide, spaced 1 cm apart. Calibration was completed according to manufacturer specifications prior to each subject’s participation and was set to record pressures between −20 and 600 mmHg (fidelity = 2 mmHg, sampling rate = 50 Hz). Protective sheaths were used to ensure sterility prior to use (ManoShield, Medtronic). Videofluoroscopy was also performed simultaneously with HRM for volitional swallows to obtain C2-C4 size. Participants were seated upright, and videos were recorded in the lateral plane at 30 frames per second (Axiom Sireskop SD, Siemens, Munich, Germany or OEC 9900, General Electric, Fairfield, Connecticut). All videos were stored electronically for offline analysis (DVO-1000MD, Sony, Park Ridge, New Jersey).

### Procedure

Volitional and non-cued saliva swallowing data for all individuals were retrospectively obtained from a research database maintained by the University of Wisconsin-Madison. Pressure and duration data for both types of swallows were analyzed using WiscMano [35], the Wisconsin Swallow Manometry protocol, which provides a standardized method for analyzing high-resolution manometry data in swallowing research. Prior to the study, patients were asked to refrain from eating and drinking for 2–4 h. During the study, *volitional* swallow data was obtained by delivering 2 cc of thin liquid barium (Varibar Thin Liquid, Bracco) to the oral cavity via syringe and cueing patient to swallow. All swallows five seconds or greater following or preceding the volitional swallow were accepted as *non-cued saliva* swallows. *Double swallows* were defined as swallows within five seconds before or after the start of the velopharynx in the cued swallow and were not considered *non-cued saliva* swallows. Other swallows were also excluded if WiscMano inaccurately identified pressure regions signified by a long “tail” in the velopharynx region, WiscMano was unable to locate standard pressure regions, a “swallowing episode” – defined as three or more consecutive swallows whose purpose is to clear the remainder of the contents in the mouth – was triggered by the cued swallow, or a non-cued swallow directly followed by a non-water bolus (e.g. lemon juice). All water boluses were delivered via syringe.

#### Outcome Variables:

In the velopharynx, tongue base, and hypopharynx regions, we obtained measurements of maximum pressure, pressure integral, and pressure duration. In the upper esophageal sphincter (UES) region, we obtained measures of maximum pre-relaxation pressure, maximum post-closure pressure, pressure integral, and nadir pressure duration. For each of the 50 participants in the study, an average over the 10 initial volitional swallows and an average over the 10 initial non-cued saliva swallows were used as the per-subject estimates for each of the measures for each swallow type within each region.

#### C2-C4 Size:

C2-C4 vertebral measurements served as a proxy for size [36, 37]. Videofluoroscopic frames were imported into ImageJ (National Institutes of Health, Bethesda, MD) for analysis. The distance between the anterior inferior corner of the C2 vertebrae and the anterior inferior corner C4 vertebrae was measured in pixels using ImageJ software. Pixel size was then converted to centimeters using the known scalar of 1 cm distance between the beginning of one manometric sensor and the next.

### Analysis

Since the primary aim of this study was to quantify differences between volitional swallows (VS) and non-cued saliva swallows (SS) in healthy adults, and the two swallow types were observed within each subject, many of our analyses were based on the VS-SS difference in pressure or duration. Thus, paired t-tests were used to assess differences between volitional and non-cued saliva swallows (referred to as delta scores throughout the text) for each pHRM parameter. Examining the impact of gender on these delta scores and C2-C4 size, we used unpaired t-tests. The association between the C2-C4 size and the pHRM pressure and duration measures and delta scores for each pHRM parameter was evaluated using Pearson’s correlation coefficient. All analyses were performed with SAS statistical software version 9.4 (SAS Institute, Inc., Cary NC). A significance level of α = 0.05 was used for all tests.

## Results

### Velopharynx

**Fig. 1:**
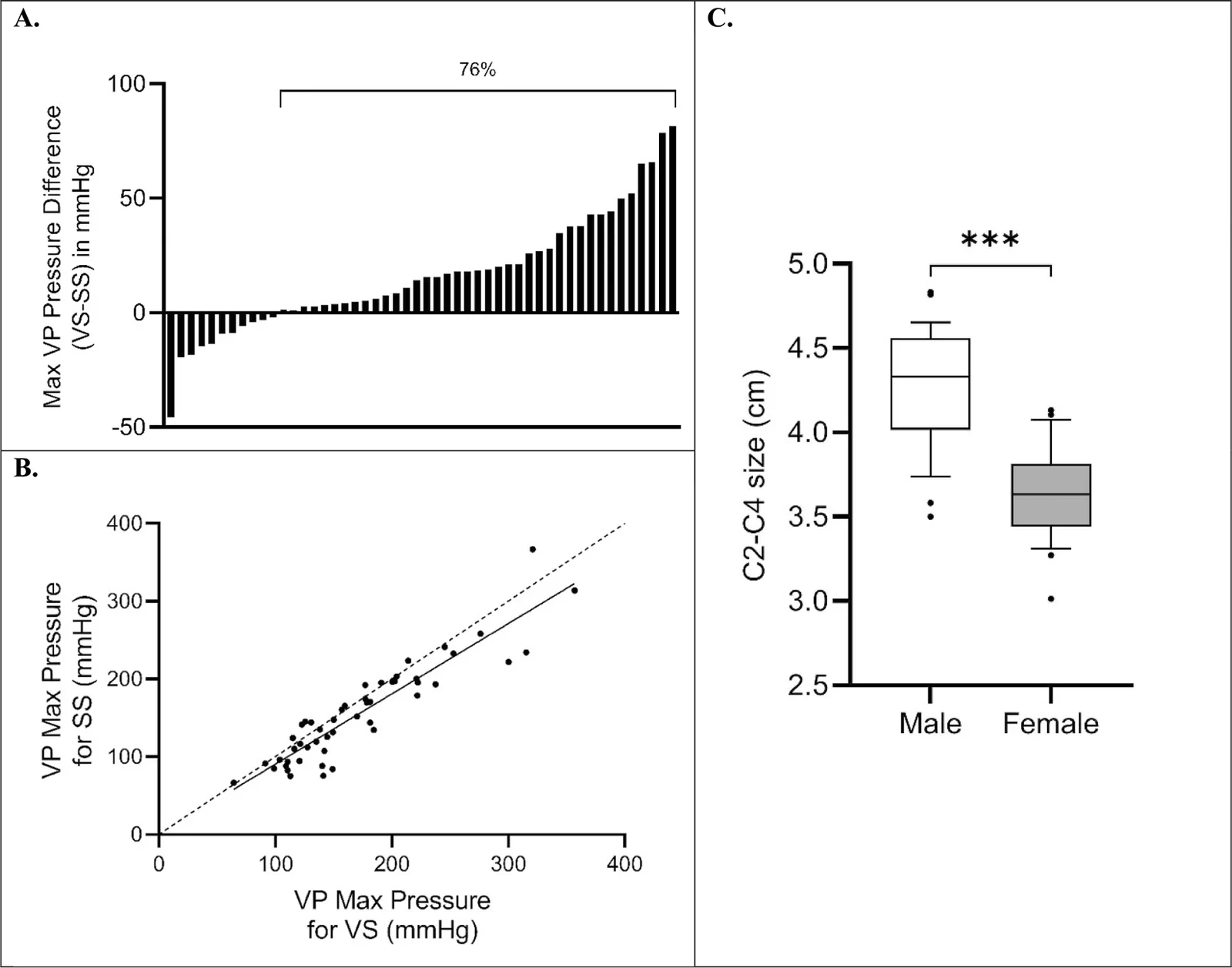
Velopharynx. (**A**) Frequency data for maximum velopharyngeal pressure. 76% of participants showed a positive pressure difference, meaning greater VS max pressures than SS max pressures in the velopharynx. (**B**) Positive relationship between velopharyngeal maximum pressures generated for volitional and non-cued saliva swallows. Line of best fit, represented by solid line, has slope of 0.9 and r^2^ of 0.846. Dashed line is included for reference of where VS and SS are exactly the same. Points below the dashed line represent subjects where VS > SS pressure; points above the dashed line represent subjects where SS > VS. (**C**) Males (white bar) had greater C2-C4 size compared to females (grey bar). From bottom to top, box plots indicate the 10th percentile (whisker below box), the 25th percentile (lower box boundary), the median (line within the box), the 75th percentile (upper box boundary), and 90th percentile (whisker above box). Values outside the 10th and 90th percentiles are indicated by dark circles. Triple asterisk represents statistical significance (***p < 0.001). SS, non-cued saliva swallow; VP, velopharyngeal; VS, volitional swallow

Maximum velopharyngeal pressure was significantly higher during volitional swallows than non-cued saliva swallows (*t*(49) = 4.57, *p* < 0.0001, *d* = 0.65). To further describe this association, we found that 76% of participants had a maximum velopharyngeal pressure that was greater during their volitional swallow than during their non-cued saliva swallow (Fig. 1A). No significant differences between swallow types were found for velopharyngeal pressure integral (*t*(49) = 1.15, *p* = 0.2576, *d* = 0.162) or velopharyngeal pressure duration (*t*(49) = 0.25, *p* = 0.8003, *d* = 0.036). Lastly, there was a positive linear relationship between swallow types (r^2^ = 0.846; slope = 0.9; Fig. 1B) for maximum velopharyngeal pressure.

There were no significant sex differences for maximum velopharyngeal pressure (*p* = 0.3828), velopharyngeal pressure integral (*p* = 0.7393), or velopharyngeal pressure duration (*p* = 0.5509).

C2-C4 size was different between males and females (*p* < 0.0001; Fig. 1C), where on average males had larger vertebrae sizes than females. Given this finding, we performed Pearson’s Correlation Coefficient to determine whether there was an association between C2-C4 size and delta scores. There were no meaningful associations between size and any of the delta scores (r < 0.1).

### Tongue Base

**Fig. 2:**
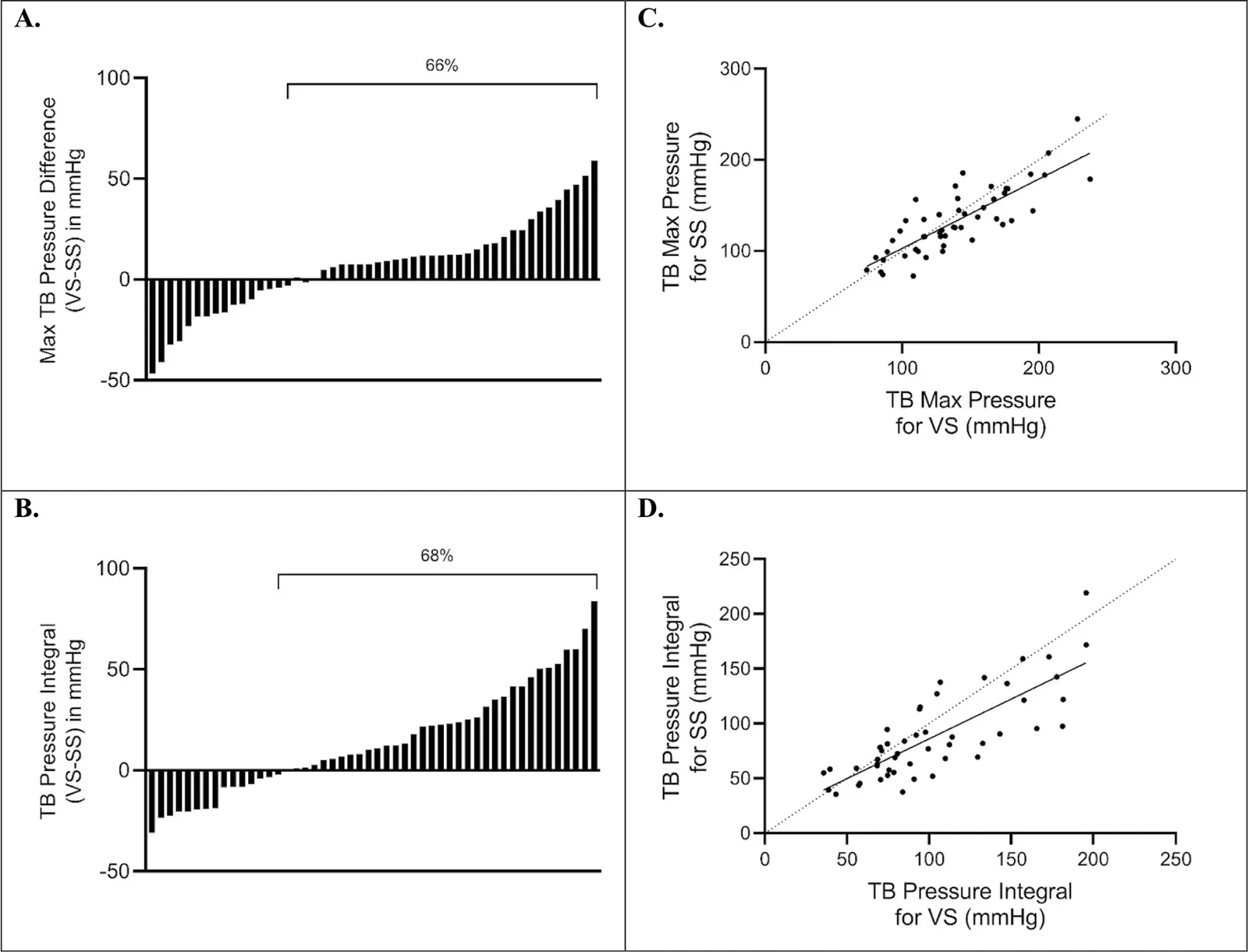
Tongue Base. (**A**) Frequency data for maximum tongue base pressure. 66% of participants showed a positive pressure difference, meaning greater VS max pressures than SS max pressures in the tongue base. (**B**) Frequency data for tongue base pressure integral 68% of participants showed a positive pressure difference, meaning. greater VS pressure integrals than SS pressure integrals in the tongue base. (**C**) Positive relationship between tongue base maximum pressures generated for volitional and non-cued saliva swallows. Line of best fit has slope of 0.76 and r^2^ of 0.67. (**D**) Positive relationship between tongue base pressure integral generated for volitional and non-cued saliva swallows. Line of best fit has slope of 0.73 and r^2^ of 0.64. For both (**C**) and (**D**) the dashed line is included for reference of where VS and SS are exactly the same. Points below the dashed line represent subjects where VS > SS pressure; points above the dashed line represent subjects where SS > VS. SS, non-cued saliva swallow; TB, tongue base; VS, volitional swallow

Maximum tongue base pressures were significantly higher during volitional swallows than non-cued saliva swallows (*t*(49) = 2.0557, *p* = 0.0452, *d* = 0.2907). Similarly, for tongue base pressure integral, we observed a significant difference between swallow types, where higher values were generated during volitional swallows than non-cued saliva swallows (*t*(49) = 3.8345, *p* = 0.0004, *d* = 0.5423). No significant difference between swallow types was found for tongue base pressure duration (*t*(49) = 1.6337, *p* = 0.1087, *d* = 0.231). To further describe these associations, we found that 66% percent of participants had a maximum tongue base pressure that was greater during their volitional swallow than during their non-cued saliva swallow (Fig. 2A). Similarly, we found that 68% of participants had a tongue base pressure integral that was greater during their volitional swallow than during their non-cued saliva swallow (Fig. 2B), and 66% of participants had a tongue base pressure duration that was greater during their volitional swallow than during their non-cued saliva swallow. There was a positive linear relationship between swallow types for maximum tongue base pressure (r2 = 0.67; slope = 0.76; Fig. 2C) and tongue base pressure integral (r2 = 0.64; slope = 0.73; Fig. 2D).

Secondary analyses revealed sex was not significant for maximum tongue base pressure (*p* = 0.5653), tongue base pressure integral (*p* = 0.4818), or tongue base pressure duration (*p* = 0.6459). Additionally, Pearson’s r revealed that there are no associations between size and any of the delta scores (*p* > 0.05, r < −0.15).

### Hypopharynx

No significant differences between swallow types were observed for maximum hypopharyngeal pressure (*t*(49) = 1.1269, *p* = 0.2653, *d* = 0.1594), hypopharyngeal pressure integral (*t*(49) = 1.0436, *p* = 0.3018, *d* = 0.1476) or hypopharyngeal pressure duration (*t*(49) = 0.6327, *p* = 0.0920, *d* = 0.0895). Secondary analyses revealed sex was not significant for maximum hypopharyngeal pressure (*p* = 0.4410), hypopharyngeal pressure integral (*p* = 0.3486), or hypopharyngeal pressure duration (*p* = 0.7865). Additionally, Pearson’s r revealed that there are no associations between size and any of the delta scores (r < −0.09).

### Upper Esophageal Sphincter

**Fig. 3:**
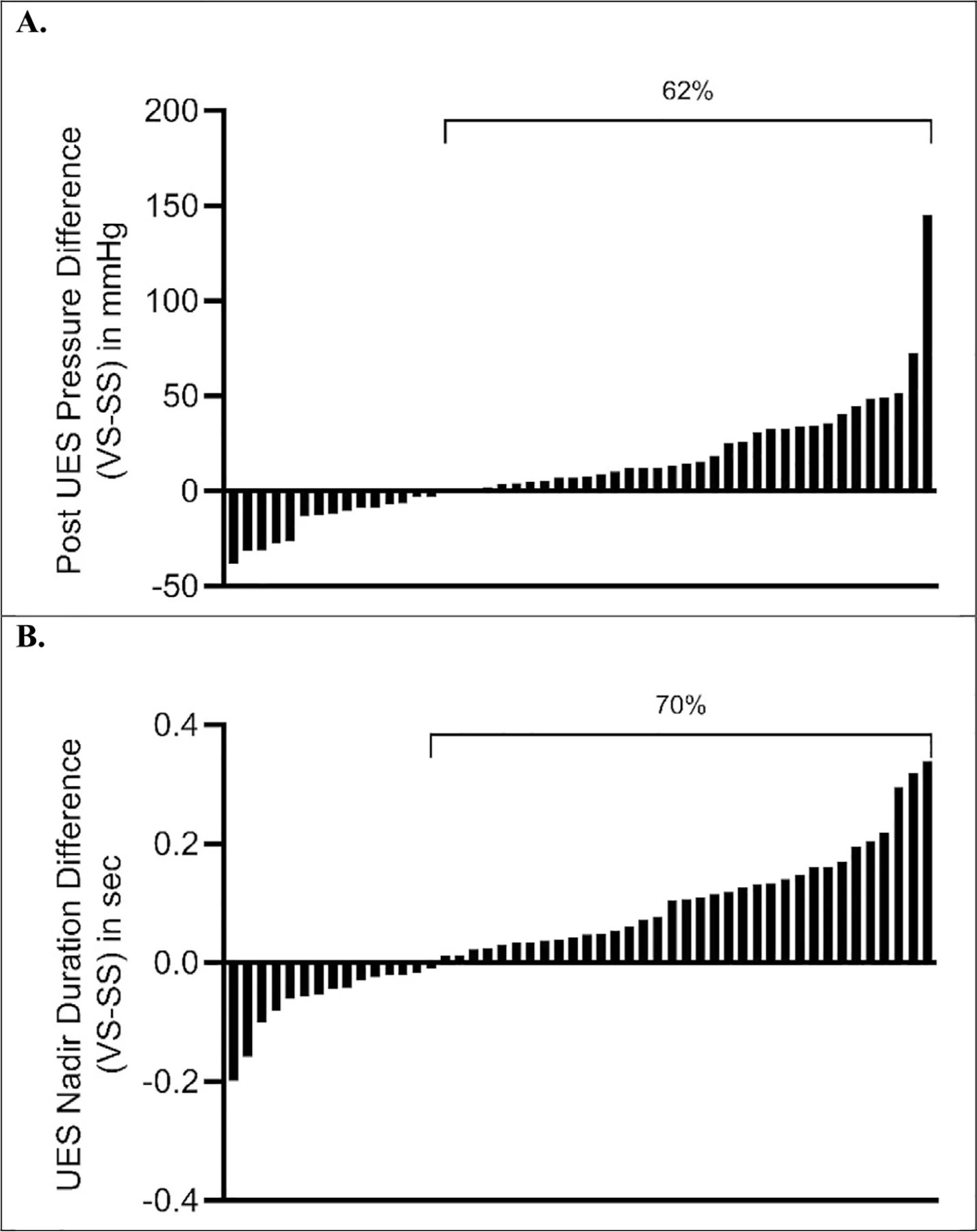
Upper Esophageal Sphincter. (**A**) Frequency data for maximum UES post-closure pressures. 62% of participants showed a positive pressure difference, meaning greater VS pressures than SS pressures post-UES closure. (**B**) Frequency data for UES nadir pressure duration. 70% of participants showed a positive duration difference, meaning longer VS duration than SS duration for UES nadir pressure. SS, non-cued saliva swallow; VS, volitional swallow; UES, upper esophageal sphincter

No significant differences between swallow types were observed for maximum UES pre-relaxation pressure (*t*(49) = −1.3436, *p* = 0.1853, *d* = −0.19) or minimum UES pressure (*t*(49) = −0.0589, *p* = 0.9532, *d* = −0.0083). However, differences between swallow types were observed for maximum UES post-closure pressure. Specifically, higher values were generated during volitional swallows than non-cued saliva swallows (*t*(49) = 2.8856, *p* = 0.0058, *d* = 0.4081). Differences were also observed for UES nadir pressure duration, where volitional swallows had greater relaxation durations than non-cued saliva swallows (*t*(49) = 3.8454, *p* = 0.0003, *d* = 0.5438). To further describe these associations, we found that 62% of participants showed greater maximum UES post-closure pressure for volitional swallows rather than non-cued saliva swallows (Fig. 3A). Furthermore, 70% of participants had a UES nadir pressure duration that was greater during their volitional swallow than during their non-cued saliva swallow (Fig. 3B). There was a weak positive linear relationship between swallow types for UES nadir pressure duration (r2 = 0.31; slope = 0.47).

Secondary analyses revealed sex was not significant for maximum UES pre-relaxation pressure (*p* = 0.1463), minimum UES pressure (*p* = 0.4551), maximum UES post-closure pressure (*p* = 0.7360), or UES nadir pressure duration (*p* = 0.7323). Additionally, Pearson’s r revealed that there are no associations between size and any of the delta scores (r < 0.23).

Mean values and standard deviations for volitional and non-cued saliva swallow pressures and durations across all regions are displayed in Table 1.

## Discussion

This is the first study to use pHRM to assess differences between volitional and non-cued saliva swallows in healthy adults. Differences in pressure and timing variables were seen between volitional and non-cued saliva swallows at most levels of the pharynx and UES. Sex was also examined as an independent variable and did not significantly affect pressure or timing measures. Although C2-C4 size was significantly larger in males than females, it did not influence any pressure or timing outcomes.

No significant difference in pressure generation was observed within the hypopharynx. This may be related to the nature of the swallows themselves. Volitional swallows with a designated volume begin with the majority of the bolus in the anterior floor of the mouth. In contrast, non-cued saliva swallows are often initiated to clear saliva that has collected in the pyriforms (in the hypopharyngeal region). This difference could change the magnitude of hyoid elevation/excursion required to transfer the bolus completely into the esophagus and maintain a safe airway. Additionally, structures in the velopharynx (*i.e*. soft palate) and tongue base may be under greater voluntary control compared to regions within the hypopharynx, potentially contributing to the larger pressure differences observed in superior pharyngeal regions.

Duration of UES relaxation was also shorter for non-cued saliva swallows compared to volitional swallows. This finding complements the pressure results, as greater pressure generation may be associated with prolonged UES relaxation duration. Bolus volume may also contribute to these differences, as the standardized 2 mL boluses used during volitional swallows likely required longer UES relaxation than the smaller and more variable volumes associated with saliva clearance swallows.

For outcomes demonstrating statistical differences between swallow types, positive relationships were observed between volitional and non-cued saliva swallow pressures and durations. In particular, very strong correlations within the velopharynx and moderate-to-strong correlations within the tongue base suggest that individuals exhibit relatively stable pressure-generating patterns across swallow types in these regions. These findings may have implications for interpretation of pHRM data within the context of current International PHRMI Working Group recommendations. The Leuven Consensus recommends assessment of pharyngeal contractility using either dry or bolus swallows, reflecting the relative stability of pharyngeal contractile function across swallow conditions [38]. The present findings further support this concept and suggest that non-cued saliva clearance swallows may provide additional information regarding pharyngeal contractility when standard bolus swallows are difficult to obtain. However, caution is warranted when interpreting UES-related metrics, as swallow volume is known to influence UES opening and relaxation characteristics. Consistent with current recommendations, larger bolus volumes remain important for comprehensive assessment of UES function and dysfunction.

The observed correlations between swallow types also suggest thar volitional swallowing performance may provide insight into spontaneous non-cued saliva swallowing performance. This may be clinically useful when evaluating patients who are NPO, have difficulty with bolus management, or have difficulty with volitional swallowing due to a number of conditions. Furthermore, interventions targeting volitional swallow performance may influence spontaneous swallowing behaviors, including secretion management, though this relationship warrants further investigation.

Although group-level analyses demonstrated higher pressures during volitional swallows, variability across individuals was also observed. Approximately half of participants demonstrated higher non-cued saliva swallow pressures in at least one region. This variability likely reflects normal physiologic differences in saliva volume, secretion characteristics, sensory state, swallowing effort, and participant comfort during testing. Similar variability has been described normative swallowing studies and may also be influenced by factors such as dry mouth [39, 40] and saliva viscosity.

Several limitations should be considered. First, the saliva swallows analyzed in this study occurred naturally throughout the swallow evaluation, and thus the exact reason for swallow initiation at non-cued times is unknown. Although these swallows functioned as spontaneous non-cued saliva swallows, it cannot be established whether they were generated solely through reflexive brainstem-mediated mechanisms or whether some degree of cortical involvement was present. Therefore, findings should be interpreted as comparisons between non-cued saliva swallows and cued 2 mL volitional swallows rather than as direct comparisons between purely reflexive and volitional swallowing. Future studies designed specifically to characterize spontaneous swallowing under controlled conditions may help further distinguish these mechanisms, although important methodological challenges remain.

Another limitation is that saliva volume during non-cued saliva swallows could not be objectively measured. Because saliva swallows are often less than 2 mL, this volume was chosen for volitional swallows, as well [41]. No additional bolus volumes were assessed despite evidence demonstrating that bolus volume significantly influences pressure and timing measures [1, 42, 43]. Future studies examining multiple volumes may provide valuable insight into the differences between spontaneous and volitional swallows, though additional instrumental methods may need to be used to control saliva volume. At present, commonly used imaging techniques such as videofluoroscopy and endoscopy do not allow objective measurement of spontaneous saliva volume, representing an important limitation for future studies overall.

Assessment of saliva (clearance) swallows should be considered in future studies given its potential utility for better understanding health and disease. Conditions such as Parkinson disease are associated with decreased swallow frequency, residue, and impaired triggering of swallows [44–47]. Thus, characterization of spontaneous swallowing may improve understanding of secretion management and swallowing efficiency and safety in populations at increased risk for residue, penetration, and aspiration [44, 48, 49]. Although this is a first attempt at understanding how saliva swallows differ from measured, cued swallows using pHRM, these findings may inform future investigations across disease states.

Lastly, larger C2-C4 vertebrae size was observed in males than females, consistent with prior literature. Despite this anatomical difference, sex was not associated with any of the 13 swallow outcomes, and Pearson’s correlations demonstrated poor relationships between cervical size and swallow measures. These findings align with prior research demonstrating that although males typically exhibit larger cervical vertebrae, these differences do not appear to substantially influence HRM outcomes [37]. Consideration of cervical size remains important when examining swallow kinematics and disease-related changes to the cervical spine, but these findings suggest that cervical size alone does not explain variability in pHRM measures [36, 50, 51].

Overall, healthy adults demonstrate greater pressures during volitional than non-cued saliva swallows, irrespective of sex. Volitional swallows appear to yield greater pressures within the velopharynx, tongue base, and post-closure UES, while nadir UES pressure durations are longer during volitional swallows. The opposite, however, was revealed to not be uncommon either and highlights the variability that exists in the healthy swallowing mechanism. The observed relationships between swallow types support current concepts regarding the relative stability of pharyngeal contractile function across swallowing conditions and suggest that non-cued saliva clearance swallows may provide clinically relevant physiologic information when traditional bolus swallows are difficult to obtain. These findings increase our understanding of swallowing physiology and may serve as foundational for refining clinical evaluations that take both volitional and spontaneous swallowing into account.

## Figures and Tables

**Table 1:** Swallow pressure and duration measures for volitional and non-cued saliva swallows

	VS	SS
Maximum velopharyngeal pressure	172.50 (64.55)	155.92 (63.45)
Velopharyngeal pressure integral	118.25 (50.76)	112.61 (56.20)
Velopharyngeal pressure duration	0.72 (0.13)	0.71 (0.18)
Maximum tongue base pressure	139.16 (38.99)	132.55 (36.19)
Tongue base pressure integral	102.08 (43.52)	87.59 (39.58)
Tongue base pressure duration	0.54 (0.10)	0.52 (0.08)
Maximum hypopharyngeal pressure	166.99 (49.54)	159.96 (50.25)
Hypopharyngeal pressure integral	57.17 (25.26)	54.03 (27.33)
Hypopharyngeal pressure duration	0.43 (0.11)	0.43 (0.11)
Maximum UES pre-opening pressure	130.28 (55.82)	136.49 (50.89)
Maximum UES post-closure pressure	231.68 (66.78)	219.23 (63.20)
Minimum UES pressure	6.60 (10.35)	6.70 (9.19)
UES nadir pressure duration	0.44 (0.13)	0.38 (0.11)

Values are presented as mean (SD). SD, standard deviation; SS, non-cued saliva swallow; VS, volitional swallow; UES, upper esophageal sphincter.

## Data Availability

The data supporting the findings of this study are available from the corresponding author upon reasonable request.
